# Integration of Herbal Medicine in Primary Care in Israel: A Jewish-Arab Cross-Cultural Perspective

**DOI:** 10.1093/ecam/nep146

**Published:** 2011-02-14

**Authors:** Eran Ben-Arye, Efraim Lev, Yael Keshet, Elad Schiff

**Affiliations:** ^1^Complementary and Traditional Medicine Unit, Department of Family Medicine, Rappaport Faculty of Medicine, Technion-Israel Institute of Technology, Haifa and Clalit Health Services, Haifa and Western Galilee District, Haifa 35013, Israel; ^2^Department of Eretz Israel Studies and School of Public Health, University of Haifa, Haifa, Israel; ^3^Department of Sociology, Western Galilee Academic College—Bar Ilan University, Israel; ^4^Department of Internal Medicine, Bnai-Zion Hospital and Department for Complementary/Integrative Medicine, Law and Ethics, International Center for Health, Law and Ethics, Haifa University, Haifa, Israel

## Abstract

Herbal medicine is a prominent complementary and alternative medicine (CAM) modality in Israel based on the country's natural diversity and impressive cultural mosaic. In this study, we compared cross-cultural perspectives of patients attending primary care clinics in northern Israel on herbal medicine specifically and CAM generally, and the possibility of integrating them within primary care. Research assistants administered a questionnaire to consecutive patients attending seven primary care clinics. About 2184 of 3713 respondents (59%) defined themselves as Muslims, Christians or Druze (henceforth Arabs) and 1529 (41%) as Jews. Arab respondents reported more use of herbs during the previous year (35 versus 27.8% *P* = .004) and of more consultations with herbal practitioners (*P* < .0001). Druze reported the highest rate of herbal consultations (67.9%) and Ashkenazi Jews the lowest rate (45.2%). About 27.5% of respondents supported adding a herbal practitioner to their clinic's medical team if CAM were to be integrated within primary care. Both Arabs and Jews report considerable usage of herbal medicine, with Arabs using it significantly more. Cross-cultural perspectives are warranted in the study of herbal medicine use in the Arab and Jewish societies.

## 1. Background

Herbal medicine is one of the main modalities in complementary and alternative medicine (CAM) and is increasingly acknowledged due to the extensive use of herbal remedies in the general population in both the developed and the developing countries worldwide [1, 2]. In the last four decades, the concept of herbal medicine has shifted from the empirical basis of traditional schools of medicine to evidence-based research into efficacy, safety, interactions with drugs and quality control. Parallel to its scientific merit, herbal medicine is also viewed in the context of culture and tradition. Various scholars claim that the cultural context of traditional and herbal medicine should be considered ethically in any discussion of intellectual property rights [3].

Israel is a unique place for the study of herbal and traditional medicine in a cross-cultural context. The country is characterized by natural diversity due to its geographic location at the meeting point of three continents, creating significant herbal diversity of approximately 2700 plant species, of which 150 (5.5%) are recorded as endemic [4]. The historical land of Israel served as an important crossroads for international trade, thereby enriching knowledge of traditional medicine and the inventory of natural products from ancient times. Evidence of the use of medicinal plants can be found in the Bible and in later Arabic and Jewish historical sources [5, 6]. Contemporary ethno-pharmacological evidence for herbal use is evident in the eastern region of the Mediterranean in both the Jewish and Arab communities [7–14]. *In vitro* and clinical research regarding efficacy and safety [15] of herbs used in traditional Islamic and Jewish medicine is emerging and includes indigenous herbs (e.g., Hypericum triquetrifolium [16], four-herb supplement of “glucolevel” [17], wheatgrass juice [18] and others).

The population of Israel is characterized by an impressive mosaic of native-born Jews, Arabs and Jewish immigrants who came to the area in several waves of immigration from dozens of countries, each with its own unique, indigenous medical traditions. Arabs (Muslims including Bedouins, Christians and Druze) are the largest minority group in Israel (in 2007 numbering 1 400 000 people), accounting for about 20% of the country's total population [19].

Data on the extent of herbal use in Israel are limited. Goldstein et al. studied patients hospitalized in the medical wards of two hospitals in Israel and reported that 26.8% of the respondents were herbal or dietary supplement consumers [20]. Ben-Arye et al. reported the prevalence of herbal use among CAM users in two studies: one with patients with psoriasis (64%) [21] and the other with patients with cancer admitted to chemotherapy treatment (19.5%) [22]. These clinical and ethnopharmacological findings suggest that herbal medicine is a prominent CAM modality in Israel. However, these findings pertain to the general population or specific ethnic groups. There is paucity in cross-cultural data that can provide a comparative analysis of herbal medicine use and patient perceptions in the diverse sub-populations in Israel. Such data are vital for any future initiative to integrate CAM with conventional care.

In this study, we assessed CAM and herbal use in patients attending primary care clinics serving people of diverse religious and cultural backgrounds in northern Israel. We hypothesized that primary care clinics may be an important setting for a future initiative to integrate CAM and conventional medicine. We aimed to explore how patients from various Israeli societies envision the possibility of adding herbal medicine practitioners to the primary care team in their local clinics.

## 2. Methods

### 2.1. Study Sites and Participants

The study was performed with a convenience sample of patients visiting primary care physicians for medical or administrative service. Participants had to be older than 18 years and medically insured by Clalit Health Services (CHS: the largest healthcare organization in Israel). The study covered seven family medicine clinics operated by the CHS in various urban and rural settings in northern Israel and serving a variety of Jewish and Arab (Muslims, Christians, Druze) populations. Prior to initiation, the study was reviewed and approved by the CHS Internal Review Board.

### 2.2. Study Design

A preliminary questionnaire in Hebrew was developed based on a comprehensive literature review as well as on a focus group discussion with patients attending a primary care clinic. A refinement of the questionnaire was conducted on the basis of the focus group's appraisals and of discussions among a group of physicians and CAM practitioners who had been asked to translate and collate the Hebrew and Arabic versions of the questionnaires bi-directionally.

The authors decided to use a broad and comprehensible definition of CAM: “therapies often named alternative, complementary, natural, folk/traditional medicine, which are not usually offered as part of the medical treatment in the clinic”. Added to this definition was a list of CAM modalities: herbal medicine, Chinese medicine (including acupuncture), homeopathy, folk, and traditional medicine, dietary/nutritional therapy (including nutritional supplements), chiropractic, movement/manual healing therapies (massage, reflexology, yoga, Alexander, and Feldenkreis techniques, etc.), mind-body techniques (meditation, guided imagery, relaxation), energy and healing therapies and other naturopathic therapies.

Research assistants administered the survey to patients attending the family clinics in 2005 and 2006. Hebrew- and Arabic-speaking research assistants were available at all of the clinics. Research assistants entered the survey data into a computer database for further analysis.

### 2.3. Data Analysis

Data were evaluated using the SPSS software program (version 12; SPSS Inc., Chicago, IL). Pearson's Chi-square test and Fisher's exact test were used to detect differences in the prevalence of categorical variables and demographic data between the Arab and Jewish participants. Also, a *t*-test was performed to detect differences in the continuous variables between the two groups. *P*-values less than .05 were regarded as significant. Chi-square tests were used to assess univariate associations with the odds ratio of CAM use in the Arab and Jewish populations.

## 3. Results

**Figure 1:**
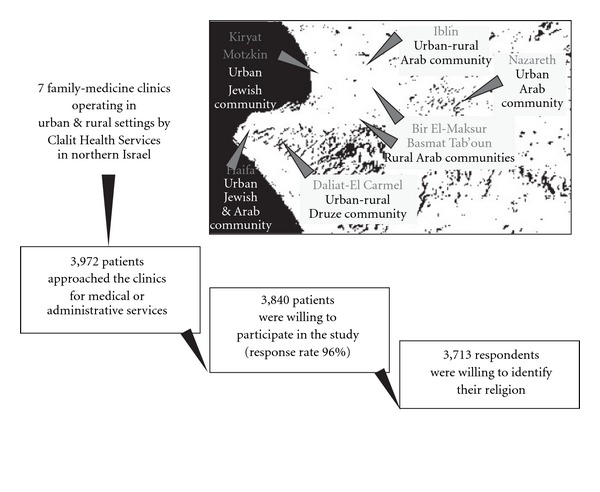
Flow diagram of recruitment in the study.

Participation in the study was offered to 3972 patients who came to seven clinics for medical or administrative services (Figure 1). Of the 3972 eligible subjects, 132 refused to participate (response rate 96%). Of the 3713 respondents who were willing to identify their religion, 2184 (59%) defined themselves as Muslims, Christians or Druze (henceforth Arabs) and 1529 (41%) as Jews.  Table 1 shows the participants' demographic characteristics, including the three Arab groups categorized according to religion (Muslims, Christians, Druze) and four Jewish social groups within the Jewish society (Israeli-born, non-native-born Ashkenazi Jews, non-native born Sephardic Jews and immigrants from the former USSR after 1990). Arab and Jewish respondents were equally distributed by sex, but differed significantly by age and education.

### 3.1. Herbal Medicine and Overall CAM Use during the Previous Year

**Figure 2:**
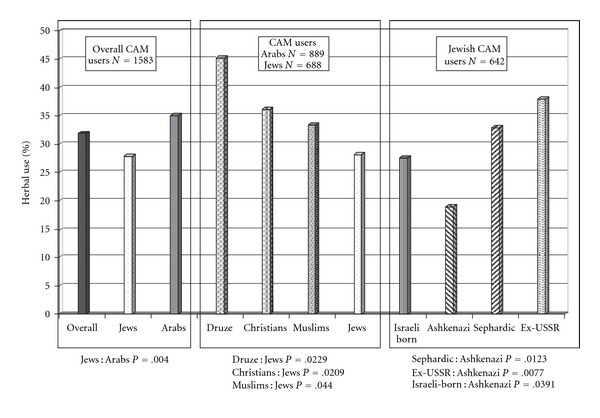
CAM users' self-reports on herbal use in the previous year.

Overall CAM use during the previous year was reported by 1583 of the 3693 respondents (42.9% CAM use). Herbal use was reported by 503 respondents (31.9% of CAM users) and was ranked fourth in prevalence out of nine CAM modalities, preceded by traditional medicine (40%), touch and manual therapies (39.7%) and nutritional supplements (37.8%). Arab respondents reported more use of herbs during the previous year than Jewish respondents (35 versus 27.8% *P* = .004), although overall CAM use was similar (Arabs 42.3%, Jews 45.6%, *P* = .06).  Figure 2 shows sub-analysis of herbal use within the Jewish and Arab groups. Jews used significantly fewer herbs (28.2%) than each of the three Arab sub-groups of Muslims (33.4% *P* = .044), Christians (36.1% *P* = .0209) and Druze (45.2% *P* = .0229). Differences in herbal use among the three Arab groups were not significant. Among the Jewish respondents, non-native-born Ashkenazi Jews reported the lowest rate of herbal use (19%), as compared with non-native born Sephardic Jews (32.9% *P* = .0123), immigrants from the former USSR after 1990 (38% *P* = .0077) and Israeli-born Jews (27.5% *P* = .0391).

### 3.2. Patients' Consultations with Herbal and Other CAM Providers

**Figure 3:**
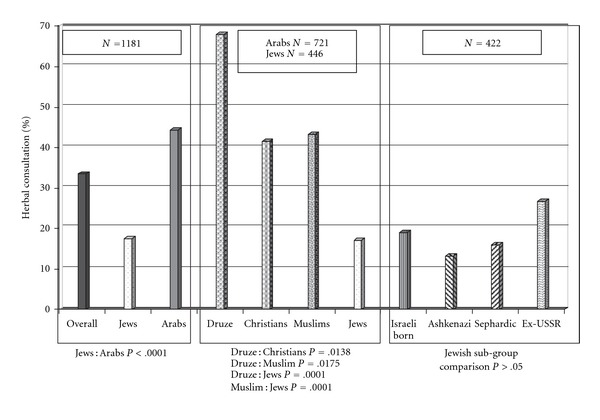
Patients' self-reports on consultations with herbal practitioners in the previous year.

Overall, 391 of 1181 (33.5%) of the study's CAM users reported conducting a consultation with herbal practitioner. Rate of consultation with herbal practitioners was higher than the rate of other CAM practitioners, and second only to that of touch and movement practitioners (46.5%).  Figure 3 shows that Arab respondents reported higher rates of consultation with herbal practitioners than Jewish respondents (44.4 versus 17.4%, *P* < .0001). Sub-analysis of herbal consultations in the Jewish and Arab groups shows that Druze reported higher referral rates to herbal practitioners (67.9%) than Muslims (43.2%, *P* = .0175), Christians (41.6%, *P* = .0138), and Jews (17%, *P* = .0001). Jews referred significantly less to herbal practitioners than Muslims (*P* = .0001) and Christians (*P* = .0001). The difference in herbal consultations within the Jewish study group was not statistically significant.

### 3.3. Expectations about Integrating Herbal/CAM Practitioners in a Primary Care Clinic

**Figure 4:**
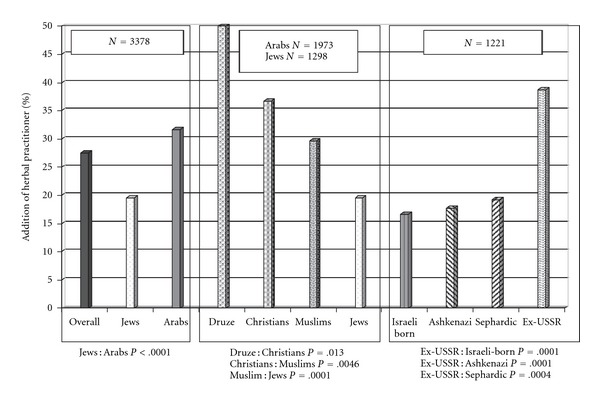
Patients' perspectives concerning addition of herbal practitioner to the primary care clinic.

Participants were asked to consider a theoretical scenario of CAM integration within their primary medical care. Overall, 3378 of the 3713 study respondents (92.8%) supported adding CAM practitioners to the clinic's medical team. Adding herbal practitioners to the clinic's team was supported by 27.5% of the respondents, more than any other option of CAM practitioners.  Figure 4 shows that Arab respondents supported adding herbal practitioners to the team more than Jews (31.6 versus 19.6%, *P* < .0001). A gradient of increasing support in this herbal practitioner option was evident in a comparison of the religious subgroups: Druze supported this option more than Christians (50 versus 36.7%, *P* = .013), Christians more than Muslims (36.7 versus 29.7%, *P* = .0046) and Muslims more than Jews (29.7 versus 19.5%, *P* = .0001). Among the Jewish subgroups, immigrants from the former USSR supported adding herbal practitioners to the clinic's team (38.7%) more than Israeli born (16.7%, *P* = .0001) and non-native Ashkenazi (17.7%, *P* = .0001) and Sephardic (19.3%, *P* = .0004) Jews.

## 4. Discussion and Conclusions

We found that herbal medicine consumption ranked fourth out of the nine CAM modalities assessed in this study, while herbal practitioner consultation ranked second. Respondents supported adding herbal practitioners to primary care clinics more than any of the other CAM practitioners. These findings may be explained in various ways: (i) safety: compared with other CAM modalities assessed in this study, herbal medicine ensures the highest safety in terms of side effects and herb-drug interactions. So, while self-use of herbal medicine is lower than other CAM modalities, patients seek more professional consultations to assure its proper and safe use. In addition, participation of a herbal practitioner in a primary care clinic may properly address the issue of herb-drug interactions. (ii) Concept: herbal medicine is easily conceptualized by patients as being similar to conventional pharmacological medicine. Hence, patients seek out a professional to prescribe herbal remedies, and the participation of an herbal practitioner in a primary care setting seems to fit, too [3]. Context: herbal medicine may be conceived by patients as a practice which is in line with conventional care; hence, it appears physically to be “working”. Patients may perceive herbs as a more drug-like modality as compared with other esoteric CAM practices considered to lie outside the conventional paradigm. However, these assumptions need validation in qualitative analysis.

Another finding was the differences in herbal use between Arabs and Jews, and within each social and religious subgroup. This difference may reflect more traditionalism in family and community structures in the rural Arab sector than in the Jewish rural sector [23]. Although over the past decades modernization has been taking place in the Arab rural and urban sectors, the gap between the Jewish and the Arab populations has not been eliminated. The differences between the two sectors in socioeconomic standards and education also carry health implications for the ongoing traditionalism in some aspects of social life, which can be seen in health-related attitudes and behaviors [24]. The extensive use of herbs among Arab respondents, as documented in our study, may reflect a more traditional attitude in the Israeli Arab society, possibly based on both a sense of connectedness to nature and tradition and more limited economic access to sufficient conventional care. In addition, Sawalha found that many Arab CAM users reported gathering herbs from nature, and suggested that CAM and herbs are popular among the Arabs because of relatedness to the local Arabic and Islamic heritage, cost considerations and accessibility, compared with modern medicine [12]. More studies are warranted to explore whether the same aspects may explain the more herbal-oriented approach that we found in our study among Sephardic Jews (mainly of North African and Asian origin) and recent Jewish immigrants from the USSR than among Israeli-born and Ashkenazi Jews (mainly of Eastern European origin).

This study does have some limitations. We did not select a representative sample of the Jewish and Arab communities in Israel but decided to approach patients in clinics serving a variety of communities with distinctive cultural characteristics that are located in a relatively small geographic area in northern Israel. This method may have caused selection bias in terms of clinic-site selection. To offset this potential bias, we made a considerable effort to minimize patient-selection bias by offering participation in the study, with no language restriction, to each and every patient who entered the clinic for any medical or administrative reason. Thus, our results may not represent the total population but rather the population of patients who actually came to the clinics. Another aspect that may limit data accuracy, and should be considered in future studies, is the appropriate way of asking patients about CAM use. Indeed, this aspect is not obvious and this is the reason why it was decided to combine in this study a set of general questions concerning use of CAM followed by questions concerning specific CAM modalities.

**Figure 5:**
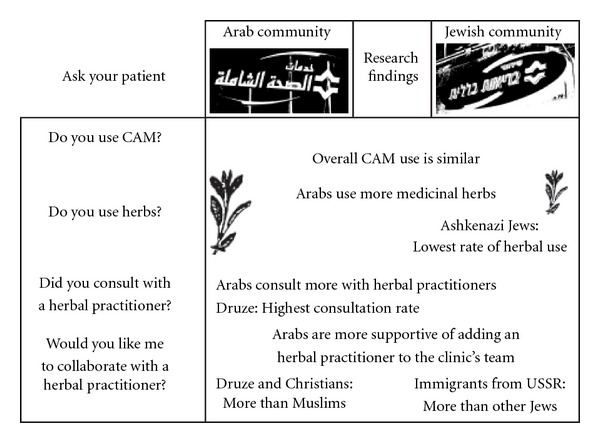
Practical implications: four questions to ask patients in primary care regarding herbs.

In conclusion, in this study we found that herbal medicine is a prominent CAM modality in northern Israel, which is subject to diverse cross-cultural attitudes in the Arab and Jewish societies. Based on our results, we recommend that primary care physicians initiate conversations with their patients regarding CAM and herbal medicine, using four simple questions (Figure 5): do you use CAM? Do you use herbs? Did you consult with an herbal practitioner? Would you like me to collaborate with the herbal practitioner? We hope this set of questions may enhance the doctor-patient dialogue, promote patient safety and improve clinical outcomes.

In addition, we recommend that future researchers take into account the limitations of our study and base their research on both qualitative and quantitative methodology, including interviews and focus group discussions in both medical and community settings.

## Figures and Tables

**Table 1:** Respondents' demographic characteristics.

Characteristic	Number of respondents who reported their religion (*n* = 3713)						
	Number of Arab respondents (*n* = 2184)			Number of Jewish respondents (*n* = 1529)^a^			
	Muslims (*n* = 1459)	Christians (*n* = 577)	Druze (*n* = 148)	Israeli-born (*n* = 742)	Ashkenazi immigrants (*n* = 353)	Sephardic immigrants (*n* = 215)	USSR immigrants (*n* = 132)
Mean age in years ± SD (median)	Arabs: 38.8 ± 13.3 (37)			Jews: 50.9 ± 17.3 (52)			
	*P* < .0001					
	Muslims	Christians	Druze	Israeli-born	“Ashkenazi”	“Sephardic”	“USSR”
	37.7 ± 12.4 (36)	41.1 ± 15.4 (38)	34.7 ± 13.4 (33)	41.7 ± 14.5 (40)	64 ± 13.8 (66)	61.3 ± 12.1 (61)	49.5 ± 17.7 (50)
	*P* < .0001 (except between Muslims and Druze)			*P* < .0001 (except between Ashkenazi and Sephardic)			

Sex, male : female (%)	Arabs:			Jews:			
	738 : 1238 (37.3 : 62.7)			548 : 883 (38.3 : 61.7)			
	Non-significant difference				
	Muslims	Christians	Druze	Israeli-born	Ashkenazi	Sephardic	USSR
	551 : 891	216 : 357	45 : 100	293 : 435	130 : 216	81 : 132	36 : 92
	(38.2 : 61.8)	(37.7 : 62.3)	(31 : 69)	(40.2 : 59.8)	(37.6 : 62.4)	(38 : 62)	(28.1 : 71.9)
	Non-significant difference			Non-significant difference except Israeli-born versus USSR immigrant			
				groups *P* = .0103			

Education:	Arabs					Jews	
1. Elementary school	317 (16.9%)			*P* = .0001		97 (7.4%)	
2. High school	1037 (55.2%)			*P* = .0015		651 (49.5%)	
3. Academic	523 (27.9%)			*P* = .0001		567 (43.1%)	
	Muslims	Christians	Druze	Israeli-born	“Ashkenazi”	“Sephardic”	“USSR”
	240 (17.7%)	88 (16.3%)	23 (17.4%)	22 (3.3%)	37 (11.8%)	38 (19.5%)	0
	783 (57.8%)	275 (50.8%)	68 (51.5%)	348 (52.3%)	143 (45.7%)	108 (55.4%)	52 (41.3%)
	331 (24.4%)	178 (32.9%)	41 (31.1%)	295 (44.4%)	133 (42.5%)	49 (25.1%)	74 (58.7%)
	Muslims compared with Christians			Israeli-born compared with other groups			
	*P* = .0056 High school			*P* < .0001 Elementary school			
	*P* = .0002 Academic studies			Sephardic compared to other groups			
				*P* < .0001 Academic studies			

SD: standard deviation. Data analysis was performed by Pearson's chi-square test and Fisher's exact test.

^a^Out of 1529 Jewish respondents, 1442 reported detailed demographic data.
